# A Cost Analysis of Implementing Asynchronous Video Directly Observed Therapy for Monitoring Treatment in Patients with Tuberculosis Disease in Uganda

**DOI:** 10.21203/rs.3.rs-7911005/v1

**Published:** 2025-11-21

**Authors:** Wilson Tumuhumbise, Rebecca Kuteesa, Damalie Nakkonde, Esther Buregyeya, Sarah Zalwango, Angella Musiimenta, Juliet N. Sekandi

**Affiliations:** Faculty of Computing and Informatics, Mbarara University of Science and Technology, Mbarara, Uganda, African Health Informatics Research Institute, Digital Health Division, Entebbe, Uganda; School of Public Health, Makerere University, Kampala, Uganda; School of Public Health, Makerere University, Kampala, Uganda; School of Public Health, Makerere University, Kampala, Uganda; School of Public Health, Makerere University, Kampala, Uganda, Kampala Capital City Authority, Department of Public Health Service and Environment, Kampala, Uganda; Faculty of Computing and Informatics, Mbarara University of Science and Technology, Mbarara, Uganda; Department of Epidemiology and Biostatistics, College of Public Health, University of Georgia, Athens, GA/USA, Global Health Institute, College of Public Health, University of Georgia, Athens, GA/USA

**Keywords:** Video Directly Observed Treatment, cost analysis, Tuberculosis, adherence, Implementation

## Abstract

**Background:**

Video directly observed treatment (VDOT) supports person-centered care and addresses shortcomings of in-person directly observed treatment (DOTS). However, there is limited literature on implementation costs to inform TB program policy in low-resource settings, such as Uganda.

**Objective:**

This study aimed to assess the costs associated with implementing the VDOT intervention in Uganda, based on a completed randomized trial.

**Methods:**

We carried out a micro-costing analysis. This employed both bottom-up and top-down approaches to estimate health system costs (in 2024 US dollars) of the VDOT intervention. The analysis covered 71 persons with TB from six health facilities in Kampala, Uganda.

**Results:**

The estimated overall costs for implementing VDOT in Uganda were $16,037.80 among 71 patients with TB. The estimated per-person costs for VDOT were $220.93. The largest proportion of the overall cost was associated with smartphones and accessories (29.5%), followed by VDOT platform infrastructure costs (21.7%), program support costs (19.5%), and internet data costs used by patients to send the videos (10.2%). The per-person costs of using VDOT are lower than the potential cost of daily transport that would be associated with facility-based DOT.

**Conclusion:**

The implementation of VDOT in Kampala was associated with a relatively low cost compared to the projected cost when using the traditional DOT method. This highlights the cost saving benefit to the patients when using remote treatment monitoring with the video technology. A full economic evaluation from the programmatic and societal perspective is recommended to inform the adoption of the VDOT strategy in resource-limited settings.

## Introduction

Tuberculosis (TB) remains a major global health concern and the leading infectious killer disease, with over 1.25 million deaths annually (1). Despite being curable, it remains the leading cause of death among individuals living with HIV/AIDS (2). In 2023 alone, about 10.8 million people fell ill from the disease globally, of which the majority were from countries in low-resource settings (LRS) (1). Uganda is among the 30 high TB/HIV burden countries with an incidence rate of 198 per 100,000 people. Treatment adherence remains a significant challenge that leads to poor TB treatment outcomes (such as treatment failure, emergence of drug-resistant TB), and secondary transmission (3, 4).

TB also places a significant economic burden on the patients and their households. In Uganda, about 53% of the households of patients with TB experience catastrophic costs between 20–40% of their entire household income, estimated at US$369 (5, 6). The World Health Organization (WHO) defines catastrophic costs as spending 20% or more of the household’s annual income on expenses related to TB care (7, 8). The largest proportion of the out-of-pocket costs are associated with travel to the TB clinics for routine visits to fill prescriptions (9). The financial burden on patients and their families hampers progress towards achieving successful TB treatment outcomes, thus negatively impacting the attainment of the End TB strategy (10, 11). The standard TB care and management strategy, known as directly observed therapy (DOT), requires daily observation of a patient as they ingest the TB medication by a health worker or treatment supporter (12). Although in-person DOT is an effective way to ensure proper adherence to treatment, its feasibility is largely limited because of a shortage of human and financial resources in the public health system (13). The resource constraints have led to the abandonment of in-person DOT in healthcare settings, rendering it less effective (14).

Digital adherence technologies (DATs) have been recommended by the WHO for addressing the shortcomings of in-person DOT and supporting person-centered TB treatment (15). These include Short Message Service, real-time medication monitoring systems, ingestible sensors, and video-observed therapy (16–19). Video directly observed therapy (VDOT) is an innovative smartphone-based system that utilizes a mobile application to record TB medication intake videos, thereby substituting the need for frequent face–to-face meetings (16) as required in the traditional DOT. The feasibility and acceptability of VDOT have been documented in studies done in TB clinics in Uganda and elsewhere (20, 21). VDOT has also been shown to be more effective in increasing medication adherence monitoring than usual care in-person DOT in an open-label randomized trial in Uganda (16).

Studies that evaluated the cost of VDOT in the United States have reported a reduction in costs of care for both patients and health departments compared to the traditional in-person DOT (22, 23). However, the cost of implementing VDOT in LRS and the associated cost savings have not been well documented. In this study, we utilized data from a recently completed VDOT trial (NCT04134689) in Uganda to conduct a secondary cost analysis.

## Methods

### Ethical Considerations

Institutional Ethical approvals for this research were obtained from the Makerere University (protocol 756) and the University of Georgia (ID PROJECT00000571) institutional review boards, and the national ethical approval from the Uganda National Council for Science and Technology (HS656ES). Written informed consent was obtained from all the study participants in either English or Luganda, depending on their language preference. Approximately US $10 was given to all the research participants per visit as compensation for transportation and the time spent participating in research activities.

### Study Setting and Participants

The study is based on a completed open-label randomized trial known as the “DOT Selfie study”, which was conducted between July 2020 and October 2021 at Lubaga TB clinic and several other public TB clinics in Kampala. The methodology of this study has been reported elsewhere (16, 24). Briefly, secondary cost data were extracted for 71 participants who were randomized to the VDOT intervention study group. Eligible patients were aged 18 to 65 years with confirmed drug-susceptible TB and residing in Kampala during the treatment period. Patients were excluded if they had confirmed multidrug-resistant TB or a documented cognitive, visual, or other disability that would interfere with recording videos. Additionally, those who did not have access to electricity to charge a smartphone or who resided in areas with poor cellular network coverage were excluded.

### Description of VDOT System

A detailed description of the VDOT system has been published elsewhere (16). Briefly, treatment adherence monitoring was performed using an asynchronous VDOT smartphone application (app) that enabled patients to record videos while swallowing each dose of medication and upload them to a secure cloud server, which was accessed and viewed later by health workers (25). The app worked on both Android (Google LLC, Menlo Park, CA, USA) and iOS (Apple Inc., Cupertino, CA, USA) smartphones. Date and time-stamped videos were automatically sent by the app through third or fourth-generation (4G) cellular networks or Wi-Fi. The app is secured to prevent videos from being viewed, edited, or deleted by users on the smartphone, thus ensuring confidentiality and fidelity of the videos. After a successful upload, the videos are automatically deleted from the smartphone. Cellular internet data was prepaid weekly at ~US$1.00 and sent directly to the participants’ phones to ensure videos would be sent in a timely manner. Trained research staff logged into an internet-based, password-protected client management system to watch the uploaded videos and documented whether they observed the pills being swallowed. The health workers followed a pre-specified protocol to follow-up with patients if a video was not received within 24 hours.

### Data Collection and Follow-up

A baseline survey was administered to collect information about participants’ socio-demographics, phone ownership, and technology use experience including using a smartphone, internet, taking photos or videos and frequency of use in the last 3 months (Supplementary Appendix 2). Additional information about different cost drivers was collected from study budget lines, receipts, time logs, Telecom Company, server, protocol specifications and cost logs. Study participants returned to the clinic for their routine monthly visit to refill prescriptions and for evaluation at 2, 4, and 6 months per standard of care.

### Costing Approaches

We adapted the VDOT costing tool guide by McGill International, published elsewhere (26) (Supplementary Appendix 1). This questionnaire aimed to capture all costs associated with the implementation of VDOT. Eight cost drivers were identified and categorized to establish detailed cost components from the health provider’s perspective, and these are; i) smart phone and accessories, ii) prepaid internet data, iii) VDOT platform/infrastructure costs, iv) program support costs (i.e. internet for viewing videos, airtime for phone communication, v) adherence monitoring by healthcare workers on VDOT platform, vi) systems and data management, vii) escalation costs related to unscheduled phone calls or home visits, and those related to VDOT technology malfunction, app upgrades or for phone repairs, and viii) VDOT training for HCWs.

At the end of the questionnaire, costs from each category were summed to estimate the total cost per patient associated with the implementation of VDOT. For costs that were only relevant to some participants, we divided them over the full patient population (i.e., prorated) in order to estimate per-patient costs.

We calculated the costs using both bottom-up (micro-costing) and top-down approaches (Cunnama 2016). Bottom-up approach focused on detailed activity and input usage data from records (time logs, cost logs, budget lines) to establish unit costs. (e.g., the smartphone and accessories cost category was established from the receipts, the cost of airtime from the time logs, to get the total cost and per-patient cost for phones and accessories).

### Program Costs

To estimate the program costs, we focused on the internet for viewing videos, airtime for phone communication, and patient follow-up as detailed activities. We then extracted the cost of internet packages from the telecom company records and time logs to establish both the patient and total cost of data bundles. For adherence monitoring by HCW on the VDOT platform,we used the fraction of time spent watching videos to confirm adherence per full-time effort of a health worker. We used time logs to establish the average time spent by research staff per patient routine and video reviewing. To establish the cost of escalation related to VDOT technology, we established the number of patients who required phone repairs, app retraining, and maintenance from the cost logs and multiplied by the average amount of costs spent on phone repairs, app retraining per participant, to get the total and per-patient cost of escalation related to VDOT technology. To estimate the cost for VDOT training for HCWs, we assessed the research assistants, their total stipulated time for training, and the hourly wage from the protocol. We then multiplied the total training time and hourly wage to get the cost of training all research assistants. To get the cost of training one research assistant for an individual patient, we established the cost of training one research assistant divided by the number of study participants.

The top-bottom approach focused on the overall expenditure (e.g., VDOT platform expenditure), then allocated costs (e.g., software license, configuration costs, to estimate costs per person, and the fixed cost of the platform. To ascertain the cost of the VDOT system and data management, we captured the fraction of time, alongside wages spent on staff providing technical support on the VDOT platform. We established the average wage per hour of the data manager and the total days spent providing the technical service to get the per-patient and total cost of this category. Escalation was related to unscheduled phone calls or home visits. We established the average wage (per hour) for research staff as per the protocol. We then established the average number of phone calls made by the health workers from call logs multiplied by the number of patients that required the phone call and or home visit support to get the per patient cost of escalation related to unscheduled phone calls or home visits.

To assess the cost savings in transportation for patients in VDOT, we looked at the average expected videos per patient. We assumed that every video was equivalent to one round trip to the TB clinic. For computation, we established the adjusted projected cost of travel over the duration of treatment by multiplying the actual days of travel to the clinic by the total cost of daily travel. To ascertain the adjusted project cost on VDOT over the duration of treatment, we considered the average per-patient cost of VDOT as a program, i.e., the summation of all cost drivers of VDOT as a program for each of the 71 participants.

### Hypothetical, Real-life, and VDOT Scenarios of Costs Associated with Travel

To illustrate the cost savings from travel, we evaluated three scenarios that were based on the two-month intensive phase of TB treatment. First, the ‘hypothetical scenario’ represents an ideal situation where there are no missed days of DOT. We assumed that a typical patient with TB who undergoes directly observed treatment at the health facility for five days a week. The patient is expected to incur round trip travel costs to the health facility for 20-week days (excluding weekends) per month without missing any schedule visit. Second, the ‘real-life scenario’ represents a situation that accounts for missed DOT days. We used the information from video submissions as equivalent to in-person hospital visits. For example, we considered the number of videos missed out of the two-month intensive phase of treatment to represent potential DOT face-to-face visits that would have been missed. Since the participants would have missed the visits, the associated travel costs were avoided—resulting in a coincidental savings. Third, the ‘VDOT-aided’ scenario represents a situation where the patient’s treatment adherence is monitored remotely by healthcare workers using digital adherence technology. This approach eliminated the need for daily travel, thereby resulting in a substantial cost saving. The only travel costs that are incurred by the patients are associated with the trips for the initial visit, intensive phase (visit one), and continuation phase (visits two & three). In all these three scenarios, we consider a constant baseline cost for the initial hospital visit for drug initiation and the monthly travel costs (20 days, excluding weekends). Therefore, the ultimate cost savings in transportation for patients in VDOT was the total costs incurred in Hypothetical ideal situation minus the total costs incurred in VDOT aided situation.

## Results

### Participants’ Baseline Characteristics

Of the 72 participants who had active TB, 50% were male, and the median age was 29.5 (24–42) years. The majority of the participants, 68 (96%), owned a cell phone, of which only 50 (70%) owned smart phones. The study loaned 21 smart phones to the 30% participants who did not own a smart phone. All 72 (100%) participants received the VDOT app except for one participant who was randomized to VDOT but refused to initiate his TB treatment soon after enrollment. The participant was included in the baseline analysis but excluded from any further analyses because he did not have the study outcomes of interest, as shown in Table 1 below.

### Cost Analysis of VDOT for 71 Participants

Our analysis categorized eight main cost drivers as shown in Fig. 1 and Table 2 below. These were further analyzed to establish both per-patient cost and the total costs incurred in the respective cost category. The largest proportion of the total cost was generated by the smartphones and accessories (31.9%), followed by the upfront cost of the VDOT platform (21.8%) and program support costs (19.6%). Others included prepaid internet subscription used by patients to submit their videos (10.2%), escalation costs for follow-up of patients (10%), of which 7.2% were related to unscheduled phone calls or home visits, while 3.8% were related to VDOT technology malfunction, app upgrade, or phone repairs. The lowest costs were related to adherence monitoring by HCWs on the VDOT platform costs (2.6%); VDOT systems and data management costs (2.3%), and the costs related to human resources training in using VDOT (0.2%).

### Projected Cost Savings from Patients’ Travel to the Health Facility over 6 months

Illustration of the cost analysis in three scenarios based on the two-month intensive phase of treatment:

### The Hypothetical Situation with no Missed In-person DOT Days

Based on the cost data collected from participants, the average cost of a daily round trip at baseline was UGX 11,450.70 ($3.18). For 71 participants, the cumulative total cost for the initial visit to the clinic was UGX 813,000 ($226) — these costs are the same in all three scenarios. To attend in-person DOT services daily for the first 20 weekdays of the first month, the per-patient average cost of travel would be UGX 229,014.10 ($63.62). Over a six-month treatment period, each participant would require an average of UGX 1,374,084.60 ($381.69) to cover daily travel costs. Including the baseline visit, the total average cost per participant for the entire treatment period would be UGX 1,385,535.30 ($384.88). Therefore, for 71 participants, the total estimated travel cost would be UGX 98,373,000 ($27,325.83), as shown in Table 3 below.

### The Real-Life Scenario Accounting for Missed In-Person DOT Days

In this scenario, we not only accounted for weekends but also for missed days of observation. A total of 859 videos were missed. During the intensive phase (visit one), each participant would incur an average travel cost of UGX 406,802 ($113), in the continuation phase, UGX 425,605.60 ($118.20) at visit two, and UGX 415,971.80 ($115.50) at visit three. In total, each participant would incur an average of UGX 1,259,830.10 ($350) for the entire treatment period. For all 71 participants, the total estimated travel cost would be UGX 89,448,000 ($24,847).

### VDOT Aided Scenario

In this scenario, we considered costs for the round-trip travel for the baseline visit UGX 11,450.70 ($3.18) and the three subsequent medication refill visits, as opposed to the 40 days of travel required in a traditional in-person DOT setup. Therefore, each participant would incur a total average of UGX 45,802.80 ($12.72) over the treatment period. For all 71 participants, the total cost was UGX 3,252,000 ($904).

## Discussion

We performed a cost analysis for the implementation of the VDOT for monitoring treatment among patients with TB to inform future adoption and potential scale up of digital adherence technologies in Uganda and similar settings. To our knowledge, this is the first study to assess the costs of a VDOT intervention among patients with drug-susceptible TB in Uganda. Our findings show that VDOT can offer a cost-saving alternative for monitoring TB treatment in this setting. The overall per-person cost of $220.93 over the 6 months of treatment was estimated for implementing VDOT in Uganda. Our findings report a slightly lower per-patient cost compared to a multi-country study that reported $304 in Moldova (n = 173), $1,154 in Haiti (n = 87), $661 in the Philippines (n = 110) (27). This is largely attributed to a relatively smaller sample considered and leveraging on participants who used their own devices, which could have reduced the costs if a study provided smartphones to all the participants. Our findings are consistent with the conclusion from a U.S based study that showed that the VDOT approach contributes to the reduction in costs of care for both patients and health departments compared to the traditional approach of direct DOT (22) and also in tandem with a study by Rosu and colleagues that reported that VDOT would reduce direct and indirect costs incurred by patients with multidrug resistant (MDR) TB by more than 90% compared to in-person DOT (28).

In our study, the largest total cost drivers were the smartphones and accessories (29.5%) and the patient costs (31.9%). This is in tandem with other global findings (27), which highlighted smartphones as a major contributor to VDOT costs. Therefore, given that VDOT is dependent on smartphones, implementers should ensure that participants have compatible devices or consider the loaning options for those who don’t have them (29). This model was also used in a study by Drabarek and colleagues in Vietnam that loaned smartphones to participants who did not own smart phones(30). Loaned smartphones increase the upfront costs of VDOT in low-resource settings (28); however, it foster a sustainable model of reusability that can be scaled up, given that different phones have different qualities, which may result in incompatibility, which affects usability (31). The smartphone loaning system has been reported to minimize the heterogeneity in quality and incompatibility of personal phone devices across patients (32). It is important to note that the cost of smartphones is gradually decreasing globally; thus the prospects of phone ownership in low income settings like Uganda will be high over time (33).

The costs related to the VDOT platform (21.7%) were another high cost driver. This is consistent with literature on expenditure of digital adherence technologies as essential one-time costs (Nsengiyumva et al. 2024). These VDOT platform costs are unavoidable should one decide to implement VDOT, for they are the lever on which the whole process is hinged. These costs are largely dependent on the existence of prior infrastructure, the ability to leverage shared cloud-based resources, and the degree of platform customization and tailoring (22, 34). However, costs can be reduced if the same platform is customized to accommodate as many participants as possible, which is dependent on the study design and the period of treatment follow-up (27).

Prepaid internet subscriptions were among the high cost drivers in this cost evaluation. This was because an internet subscription is required by both patients and healthcare providers for the VDOT program implementation. This is consistent with what has been reported in other settings (34) and our previous study that reported internet costs as a potential barrier to the uptake of VDOT in Uganda (35, 36). In the future, the National TB scale-up programs should explore public-private partnerships with telecommunications companies. These partnerships could, in turn, allocate corporate social responsibility resources to subsidize internet data costs. However, with the new advances in internet technology like 5G and 6G that enhance high data communication rates, low latency, and reliability (37), access to the internet is projected to expand in LRS like Uganda. On the other hand, developers should think of standalone applications that do not require the internet for patients to upload videos (38).

Our cost analysis shows that there is substantial cost savings in transportation associated with VDOT. For example, a patient who uses VDOT could save thirty times more ($12.72 vs $384.88) in transportation costs than if the standard in-person facility based DOT is used. This comparison assumes that a participant doesn’t miss any clinic visits for the whole treatment period. When we further compared the real life scenario where a participant may miss going to the facility for in-person DOT (considering that a missed video submission is a missed hospital visit), we found that a VDOT patient would on average still incur a total of $12.72 compared to $350 that he would have incurred in the real life scenario of in-person DOT during the entire six-month treatment period. This implies that VDOT is less costly, as confirmed by a study by Lam and colleagues that reported a significant cost saving while using VDOT (39). Therefore, considering both travel costs and per-patient costs associated with the implementation of the VDOT in this setting (e.g., internet costs, smartphone accessories, airtime) on the side of the user and platform/infrastructure costs, system/data management, and training on the side of the facility, VDOT is associated with lower implementation costs.

Our cost analysis has some strengths and limitations. This study is the first to conduct a preliminary cost evaluation of VDOT implemented in the local Uganda context; therefore, the results could inform the next steps of scale-up. We conducted a partial economic evaluation that does not account for the real cost of usual care and a full scale of societal costs; therefore, a cost-effectiveness analysis study is recommended. The secondary cost analysis is based on a limited sample size from the pilot VDOT trial conducted in an urban setting; therefore, the findings may not be generalizable to rural settings. We recommend future research that explores the costs of VDOT in both urban and rural populations, as there might be context specific differences in cost drivers.

## Conclusion

The implementation of VDOT for monitoring medication adherence among patients with drug susceptible TB was cost saving compared to a hypothetical scenario of using in-person facility-based DOT in an urban Ugandan setting. We recommend further research to evaluate the cost-effectiveness of VDOT.

## Figures and Tables

**Figure 1:** Per Patient Costs for Implementing VDOT Intervention

**Table 1:** Baseline characteristics of VDOT study participants (N = 72)

Variables	Variables	VDOT Arm (%)n = 72
**Sex**	**Sex**	
	Male	36 (50.0)
	Female	36 (50.0)
**Age in years, median (IQR)**	**Age in years, median (IQR)**	29.5 (24–42)
**Age Category**	**Age Category**	
18–24	18–24	19 (70.4)
25–34	25–34	27 (56.2)
35–44	35–44	10 (33.3)
45–65	45–65	16 (41.0)
**Highest level of education**	**Highest level of education**	
	No education or primary	27 (37.5)
	Secondary	28 (38.9)
	Tertiary/University	17 (23.6)
**Marital status**	**Marital status**	
	Currently married	23 (31.9)
	Previously married	13 (18.1)
	Never married	36 (50.0)
**Currently employed**	**Currently employed**	
	No	29 (40.3)
	Yes	43 (59.7)
**Monthlypersonal income in USD§, median (IQR)**	**Monthlypersonal income in USD§, median (IQR)**	42.85 (14.29–114.30)
**Monthly total household income in USD§, median (IQR)**	**Monthly total household income in USD§, median (IQR)**	71.43 (28.57–157.14)
	**Currently owned cellphone**	
	No	14 (9.4)
	Yes	58 (80.6)
**Sex**	**Sex**	
	**Owned smartphone**	
	No	22 (30.6)
	Yes	50 (69.4)
	**HIV Status**	
	Positive	24 (33.3)
	Negative	48 (66.7)

**Table 2:** Cost Analysis of VDOT for 71 Patients in Kampala, Uganda

Cost Category	Per Patient Cost in UGX	Per Patient Cost in USD	Per Patient Cost (%)	Total Cost in UGX	Total Cost in USD	Total Cost (%)
Smart Phone and Accessories	253,973	70.54	31.9	17,016,191	4726.72	29.5
Prepaid Internet Data	81,750	22.70	10.2	5,886,000	1635	10.2
VDOT Platform / Infrastructure Costs	173,625	48.22	21.8	12,501,120	3472.53	21.7
Program support costs (i.e. internet for viewing videos, airtime for phone communication	156,389	43.44	19.6	11,260,000	3127.78	19.5
Adherence Monitoring by HCWs on VDOT Platform	21,196.4	5.88	2.7	1,483,748	412.15	2.6
Systems and Data Management	18,374	5.10	2.3	5,659,192	1572.00	9.8
Escalation costs related to unscheduled phone calls or home visits	57,397	15.94	7.2	2,382,336	661.76	4.1
Escalation costs related to VDOT technology	30,500	8.47	3.8	610,000	169.44	1.1
VDOT training for HCWs	2,170	0.60	0.3	937,500	260.42	1.6
**Grand total**	795,374	220.93	100	57,736,087	16037.80	100

One (1) United States Dollar (USD) ~ 3600 Uganda Shillings (UGX) in 2025

HCW- Health Care Worker

**Table 3:** Cost Saving Scenarios using Participant Travel Cost Data (n = 71)

Hypothetical (Ideal) facility based DOT (no missed doses) Scenario	Hypothetical (Ideal) facility based DOT (no missed doses) Scenario	Hypothetical (Ideal) facility based DOT (no missed doses) Scenario	Hypothetical (Ideal) facility based DOT (no missed doses) Scenario	Hypothetical (Ideal) facility based DOT (no missed doses) Scenario
Treatment Period	Cumulative cost for N = 71 (UGX)	Cumulative cost for N = 71 (USD)	Mean (SD) per patient cost (UGX)	Mean (SD) per patient cost (USD)
Baseline (Cost of daily round trip per participant)	813,000	226	11450.7 (8883)	3.18 (2.5)
Monthly travel cost	16,260,000	4516.67	229,014.1 (177,660.6)	63.615 (49.4)
Travel cost for six months	97,560,000	27100.02	1,374,084.6 (1,065,963.9)	381.69 (296.1)
Total cumulative ideal costs over six months treatment period	98,373,000	27,325.83	1,385,535.3 (1074,846.9)	384.88 (298.6)
Real-world facility based DOT/ (859 missed doses) Scenario	Real-world facility based DOT/ (859 missed doses) Scenario	Real-world facility based DOT/ (859 missed doses) Scenario	Real-world facility based DOT/ (859 missed doses) Scenario	Real-world facility based DOT/ (859 missed doses) Scenario
Baseline	813,000	226	11450.7 (8883)	3.18 (2.5)
Travel cost at visit one (intensive phase— 321 videos were missed)	28,883,000	8023	406802.0 (316174)	113 (87.8)
Travel cost at visit two (Continuation phase— 236 videos were missed)	30,218,000	8394	425605.6 (339041.3)	118.2 (94.2)
Travel cost at visit three (Continuation phase— 302 videos were missed)	29,534,000	8204	415971.8 (352288.1)	115.5 (97.8)
Total cumulative real-world costs over six months treatment period	89,448,000	24847	1,259,830.1 (1,016,386.4)	350 (282)
VDOT-aided Scenario Hypothetical (Ideal) facility based DOT (no missed doses) Scenario	VDOT-aided Scenario Hypothetical (Ideal) facility based DOT (no missed doses) Scenario	VDOT-aided Scenario Hypothetical (Ideal) facility based DOT (no missed doses) Scenario	VDOT-aided Scenario Hypothetical (Ideal) facility based DOT (no missed doses) Scenario	VDOT-aided Scenario Hypothetical (Ideal) facility based DOT (no missed doses) Scenario
Treatment Period	Cumulative cost for N = 71 (UGX)	Cumulative cost for N = 71 (USD)	Mean (SD) per patient cost (UGX)	Mean (SD) per patient cost (USD)
Baseline (Cost of daily round trip per participant)	813,000	226	11450.7 (8883)	3.18 (2.5)
Monthly travel cost at each visit	813,000	226	11450.7 (8883)	3.18 (2.47)
Cumulative Travel cost over six months treatment period	3,252,000	904	45,802.8 (35,532.1)	12.72 (9.9)
Cost savings in travel for patients in VDOT	Cost savings in travel for patients in VDOT	Cost savings in travel for patients in VDOT	Cost savings in travel for patients in VDOT	Cost savings in travel for patients in VDOT
Cost saving	Cost saving	Cost saving	Total amount (UGX)	Total amount (USD)
Individual travel cost saving (Cumulative per-patient travel cost incurred in the hypothetical ideal situation - Cumulative per-patient travel cost incurred in the VDOT aided scenario)	Individual travel cost saving (Cumulative per-patient travel cost incurred in the hypothetical ideal situation - Cumulative per-patient travel cost incurred in the VDOT aided scenario)	Individual travel cost saving (Cumulative per-patient travel cost incurred in the hypothetical ideal situation - Cumulative per-patient travel cost incurred in the VDOT aided scenario)	1,339,732.5	372.14
Real beneficial cost saving (Total costs incurred in Hypothetical ideal situation - Total costs incurred in VDOT aided situation)	Real beneficial cost saving (Total costs incurred in Hypothetical ideal situation - Total costs incurred in VDOT aided situation)	Real beneficial cost saving (Total costs incurred in Hypothetical ideal situation - Total costs incurred in VDOT aided situation)	95,121,000	26,422.5
Coincidental cost saving (total missed doses * Average daily round trip cost)	Coincidental cost saving (total missed doses * Average daily round trip cost)	Coincidental cost saving (total missed doses * Average daily round trip cost)	9,836,151.3	2732.26

## Data Availability

The datasets used and/or analysed during the current study are available from the corresponding author on a reasonable request.
